# High handaxe symmetry at the beginning of the European Acheulian: The data from la Noira (France) in context

**DOI:** 10.1371/journal.pone.0177063

**Published:** 2017-05-17

**Authors:** Radu Iovita, Inbal Tuvi-Arad, Marie-Hélène Moncel, Jackie Despriée, Pierre Voinchet, Jean-Jacques Bahain

**Affiliations:** 1 MONREPOS Archaeological Research Centre and Museum for Human Behavioural Evolution, Römisch-Germanisches Zentralmuseum, Leibniz-Forschungsinstitut für Archäologie, Neuwied, Germany; 2 Center for the Study of Human Origins, Department of Anthropology, New York University, New York, United States of America; 3 Department of Natural Sciences, The Open University of Israel, Raanana, Israel; 4 Département de Préhistoire-UMR 7194 CNRS, Institut de Paléontologie Humaine, Muséum National d'Histoire Naturelle, Paris, France; Universidade do Algarve, PORTUGAL

## Abstract

In the last few decades, new discoveries have pushed the beginning of the biface-rich European Acheulian from 500 thousand years (ka) ago back to at least 700 ka, and possibly to 1 million years (Ma) ago. It remains, however, unclear to date if handaxes arrived in Europe as a fully developed technology or if they evolved locally from core-and-flake industries. This issue is also linked with another long-standing debate on the existence and behavioral, cognitive, and social meaning of a possibly chronological trend for increased handaxe symmetry throughout the Lower Paleolithic. The newly discovered sites can provide a link between the much older Acheulian in Africa and the Levant and the well-known assemblages from the later European Acheulian, enabling a rigorous testing of these hypotheses using modern morphometric methods. Here we use the Continuous Symmetry Measure (CSM) method to quantify handaxe symmetry at la Noira, a newly excavated site in central France, which features two archaeological levels, respectively ca. 700 ka and 500 ka old. In order to provide a context for the new data, we use a large aggregate from the well-known 500 ka old site of Boxgrove, England. We show that handaxes from the oldest layer at la Noira, although on average less symmetric than both those from the younger layers at the same site and than those from Boxgrove, are nevertheless much more symmetric than other early Acheulian specimens evaluated using the CSM method. We also correlate trends in symmetry to degree of reduction, demonstrating that raw material availability and discard patterns may affect observed symmetry values. We conclude that it is likely that, by the time the Acheulian arrived in Europe, its makers were, from a cognitive and motor-control point of view, already capable of producing the symmetric variant of this technology.

## Introduction

From the very beginning of their first discovery in the 19^th^ century [1], handaxes have been the subject of extraordinary fascination, a fact that has led to some very rigorous research on the objects themselves, but also to much speculation about their social and even biological meaning [2,3]. Much of this fascination has to do with our perception of these objects as *intentionally* 'well-made', 'symmetric', or indeed 'beautiful' [4], and hence, as an index of the hominins' aesthetic appreciation [5] and cognitive [6,7] abilities. The idea that handaxe symmetry and shape standardization not only serve a particular *purpose*, be it utilitarian or social-symbolic, but also that this property gradually improves with time can be traced back to de Mortillet [8] and was reprised in turn by Bordes, Roe and many others [9–12]. The usual explanation is that increasing symmetry is related to brain development and associated capacities for imposing form on natural materials–simply put, as hominins became smarter, they produced more symmetric/beautiful handaxes. Such evolutionary trajectory arguments can be found to this day in the literature on the African [13], Levantine [14], and European [15,16] Acheulian. An opposing, yet diverse collection of views, holds that such a trend is nonexistent [17,18], or that, if it exists, it reflects either non-intentional cultural practices, such as copy-error [19–21] or functional constraints on tool shape [22], life history of the tool or geometric constraints due to raw material choices [23], rather than cognitive differences.

**Fig 1:**
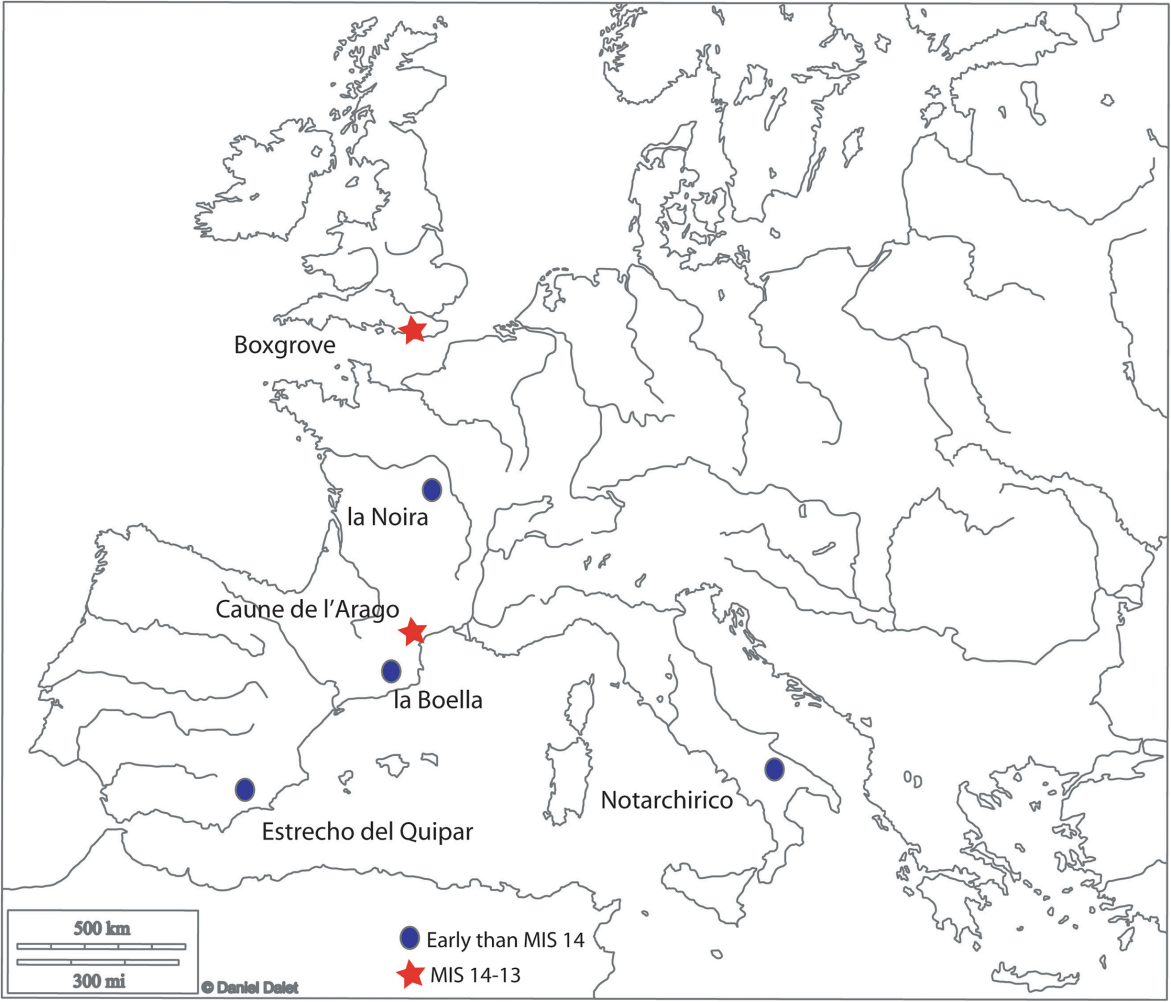
Geographic context. Map of Europe showing the location of the two sites discussed here along with that of other important sites in the relevant period.

In the last decade, new field discoveries and analyses of Acheulian assemblages have shown the diversity of form and process involved in handaxe production, as well as the association of symmetric and asymmetric handaxes in the same series and over time [6,18,24–30]. Lithic data from the Early Acheulian in Africa indicate a trend towards standardization and symmetry over time [13]. As mentioned above, the significance of this trend is unknown, and its existence in other areas adjacent to Africa, such as the Levant [14] and especially Western Europe, where the earliest evidence of bifacial technology emerged sometime between 700 and 500 ka, is disputed [16,18], Unfortunately, many of these competing accounts suffer from the use of subjective assessments of shape, small sample sizes, the use of different or newly invented measures of symmetry and shape quantification, or lack associated studies of raw materials, techniques, and discard patterns, or various combinations of the above. Therefore, the quantification of handaxe symmetry is a key to making comparisons among assemblages and drawing conclusions about the timing of technological innovations and skill achievements. In the present paper, we present Elliptical Fourier (EF) shape data and Continuous Symmetry Measure (CSM) data on handaxe contours from the site of Brinay la Noira (central France), contextualized by technological and raw-material studies, as well as comparative data from another key site, Boxgrove (England) (see Fig 1 below). The lower level at la Noira (ca. 700 ka) represents the earliest available statistically relevant handaxe assemblage on the entire European continent, whereas the upper level (ca. 500 ka) allows for both intra-site and external comparisons. La Noira and Boxgrove offer two windows on hominin behavior, separated in time by 200 ka. We use Boxgrove, a site highly regarded for its craftsmanship, to compare and assess the minimal observable technical ability evidenced by the La Noira handaxes. We thereby attempt to bracket the abilities of hominins at the beginning of the Acheulian in Europe to produce symmetric objects.

## Materials and methods

This paper focuses on the earliest handaxes from the lower level at la Noira, while at the same time providing a context through comparisons with the upper level at the same site and with a large aggregate of handaxes from Boxgrove, UK. The latter was selected as a comparison because it is one of the oldest and largest (in terms of sample size) handaxe assemblages in Eurasia that is made on fine-grained, easy to knap siliceous stone. Both la Noira and Boxgrove are located relatively close to their respective sources of raw material equalizing the variable of transport. This allows us to assume that the knappers' abilities were not in any way hampered by raw material-specific issues with controlling fractures. Moreover, Boxgrove is appropriate as a measuring stick also because a large part of the handaxes are generally recognized as exhibiting a high degree of craftsmanship (in folk parlance, they are 'well-made').

### Presentation of the samples

#### La Noira

The site of la Noira is located in the Middle Loire Basin (Centre region, France, see Fig 1 below). In this area, the fossil fluvial system of the Cher River is composed of nine stepped fossil alluvial formations deposited during the Early and Middle Pleistocene. The site of la Noira is covered by the Fougères Formation, which is one of the stepped Pleistocene fossil fluvial sheets deposited by the Cher River [31–34].

Five successive strata can be observed (from bottom to top): a coarse slope deposit or diamicton (stratum a), covered by two sequences of sandy alluvial layers (stratum b), then a rubble level (stratum c) and a sandy-silty soil (stratum d). The oldest archaeological level studied in this article (henceforth referred to as the 'lower level') is located in stratum a, whereas the younger, upper level is located at the top of stratum c.

The basal layer (stratum a) was deposited on the limestone bedrock at the beginning of a glacial stage after the river incision. It is a coarse deposit downsloped from the plateau and containing numerous weathered blocks or pebbles of endogenous rocks, sedimentary rocks in a brown rubefied clayey matrix. These slope deposits also contained lacustrine millstone, which is a siliceous sedimentary rock. Hominins selected millstone slabs of various thicknesses, for knapping and shaping. The site appears to have been both a workshop and a place for domestic activities, as suggested by the documented lithic strategies and preliminary micro-wear results [29].

The age of fluvial formation was determined using the ESR method applied to optically bleached sedimentary quartz grains. The results are very coherent and the reproducibility is good. The mean ESR age value obtained for stratum b of sandy formations is 655 ± 55 ka. Tests with cosmogenic nuclide dating provide a similar value of 730 ± 210 ka but with margins of error that are too large [35]. The average age of the human occupation is thus of around 700 ka [34,36]. The hominin occupation occurred between the end of river incision and the fluvial deposits and suggests that hominins were present during the beginning of the glacial stage, just before the pleniglacial phase and before interglacial fluvial deposition. This glacial/interglacial cycle can be assigned to the MIS 16/ MIS 15 cycle, according to the ESR results obtained for the fluvial layer [27]. The upper part of the sequence (top of stratum c) is dated to 449 ± 45 ka. This stratum c had overlaid an erosive surface which truncated ice-wedges, that suggests that those artifacts were probably abandoned during a temperate phase.

Chronologically, la Noira joins recent discoveries in Spain, France, and England, which enrich our vision of the first Acheulian colonization in the southern and northern parts of Europe and attest to the onset of biface technology before 500 ka: Notarchirico (600 ka) in Italy and Arago (older than 550 ka, levels P and Q) in the South of France [25,26,28,37–39]. Moreover, the recent discovery of la Boella [40] and Estrecho del Quípar [41] in Spain suggests pushing the starting point of European bifacial technology close to the 1 Ma mark (but see [42] for a critique of the chronology). Unfortunately, unlike la Noira, all of these other assemblages are too small for a statistically significant characterization of the industry.

**Fig 2:**
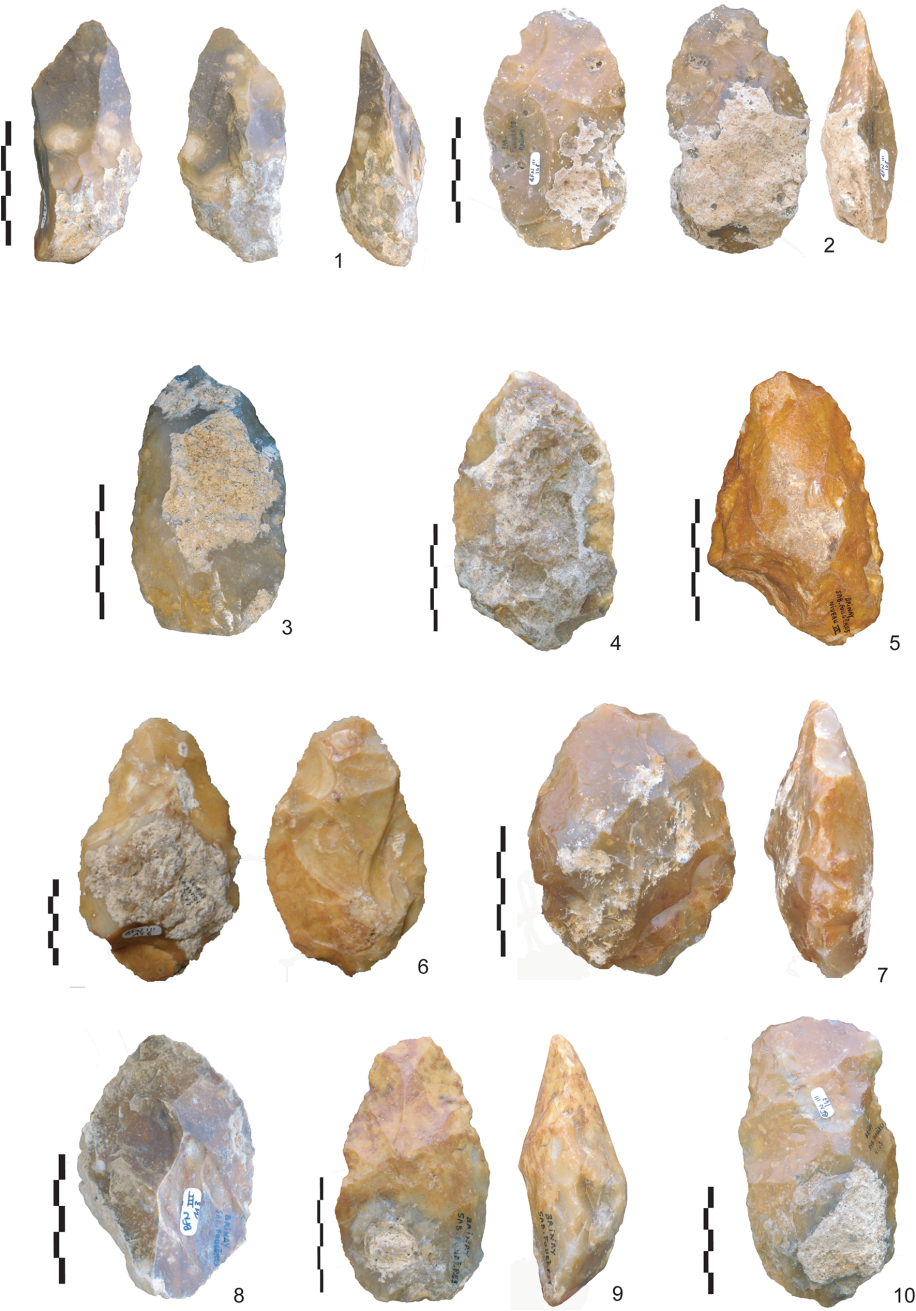
Handaxes from la Noira, lower level. Examples of the types of millstone handaxes at the lower level at la Noira: crudely-made bifacial tools (1, 2, 9, 10) bifacial tools (4), bifaces (3, 6, 7) and bifacial cleavers (8).

Therefore, la Noira represents the best currently available evidence for the occurrence of a typical Acheulian bifacial technology. The collection is located at the Institut de Paléontologie Humaine (IPH), Fondation Albert 1er, Prince de Monaco 1, rue René Panhard 75013 Paris FRANCE (iph@mnhn.fr). The following specimen numbers are contained in the collection: BFNIII-6,BFNIII-7,BFNIII-12,BFLN-13,BFNIII-15,BFNIII-16,BFNIII-22,BFNIII-23,BFNIII-32,BFNIII-39,BFNIII-44,BFNIII-49,BFNIII-68,BFLN12-80,BFNIII-81,BFNIII-87,BFNIII-108,BFNIII-109,BFNIII-110,BFNIII-112,BFNIII-113,BFNIII-120,BFNIII-142,BFNIII-145,BFNIII-146,BFNIII-147,BFNIII-148,BFNIII-150,BFNIII-155,BFNIII-157,BFNIII-260,BLNM24-1,BLNE1D2-2,BFNVIC-7,BFNVIC-11,BFNVIC-17,BFNVIC-19,BFNVIC-20,BFNVIB-21,BFNVIC-23,BFNVIB-24,BFNVIB-26,BFNVIC-28,BFNVIC-29,BFNVIC-30,BFNVIC-31,BFNVIC-32,BFNVIC-40,BFNVIC-41,BFNVIC-42,BFNVIB-44,BFNVIB-46,BFNVIC-46,BFNVIC-47,BFNVIC-62,BFNVIB-99,BFNVIB-101,BFNVIB-102,BFNVIB-103,BFNVIB-104,BFNVIB-105,BFNVIB-106,BFNVIB-107,BFNVIB-109,BFNVIB-112,BFNVIB-115,BFNVIB-116,BFNVIB-121,BFNVIB-124,BFNVIB-125,BFNVIB-133,BFNVIB-134,BFNVIB-177,BFNVIB-178,BFNVIB-179,BFNVIB-180,BFNVIB-291,BFNVIB-292,BFNVIB-303,BFNVIB-304,BFNVIB-306,BFNVIB-BEAU,BFNIII-6,BFNIII-7,BFNIII-12,BFLN-13,BFNIII-15,BFNIII-16,BFNIII-22,BFNIII-23,BFNIII-32,BFNIII-39,BFNIII-44,BFNIII-49,BFNIII-68,BFLN12-80,BFNIII-81,BFNIII-87,BFNIII-108,BFNIII-109,BFNIII-110,BFNIII-112,BFNIII-113,BFNIII-120,BFNIII-142,BFNIII-145,BFNIII-146,BFNIII-147,BFNIII-148,BFNIII-150,BFNIII-155,BFNIII-157,BFNIII-260,BLNE1D2-2,BLNM24-1,BFNVIC-7,BFNVIC-11,BFNVIC-17,BFNVIC-19,BFNVIC-20,BFNVIB-21,BFNVIC-23,BFNVIB-24,BFNVIB-26,BFNVIC-28,BFNVIC-29,BFNVIC-30,BFNVIC-31,BFNVIC-32,BFNVIC-40,BFNVIC-41,BFNVIC-42,BFNVIB-44,BFNVIB-46,BFNVIC-46,BFNVIC-47,BFNVIC-62,BFNVIB-99,BFNVIB-101,BFNVIB-102,BFNVIB-103,BFNVIB-104,BFNVIB-105,BFNVIB-106,BFNVIB-107,BFNVIB-109,BFNVIB-112,BFNVIB-115,BFNVIB-116,BFNVIB-121,BFNVIB-124,BFNVIB-125,BFNVIB-133,BFNVIB-134,BFNVIB-177,BFNVIB-178,BFNVIB-179,BFNVIB-180,BFNVIB-291,BFNVIB-292,BFNVIB-303,BFNVIB-304,BFNVIB-306,BFNVIB-BEAU,BFNIII-6,BFNIII-7,BFNIII-12,BFLN-13,BFNIII-15,BFNIII-16,BFNIII-22,BFNIII-23,BFNIII-32,BFNIII-39,BFNIII-44,BFNIII-49,BFNIII-68,BFLN12-80,BFNIII-81,BFNIII-87,BFNIII-108,BFNIII-109,BFNIII-110,BFNIII-112,BFNIII-113,BFNIII-120,BFNIII-142,BFNIII-145,BFNIII-146,BFNIII-147,BFNIII-148,BFNIII-150,BFNIII-155,BFNIII-157,BFNIII-260,BLNE1D2-2,BLNM24-1,BFNVIC-7,BFNVIC-11,BFNVIC-17,BFNVIC-19,BFNVIC-20,BFNVIB-21,BFNVIC-23,BFNVIB-24,BFNVIB-26,BFNVIC-28,BFNVIC-29,BFNVIC-30,BFNVIC-31,BFNVIC-32,BFNVIC-40,BFNVIC-41,BFNVIC-42,BFNVIB-44,BFNVIB-46,BFNVIC-46,BFNVIC-47,BFNVIC-62,BFNVIB-99,BFNVIB-101,BFNVIB-102,BFNVIB-103,BFNVIB-104,BFNVIB-105,BFNVIB-106,BFNVIB-107,BFNVIB-109,BFNVIB-112,BFNVIB-115,BFNVIB-116,BFNVIB-121,BFNVIB-124,BFNVIB-125,BFNVIB-133,BFNVIB-134,BFNVIB-177,BFNVIB-178,BFNVIB-179,BFNVIB-180,BFNVIB-291,BFNVIB-292,BFNVIB-303,BFNVIB-304,BFNVIB-306,BFNVIB-BEAU). The assemblage features bifaces, bifacial tools, bifacial cleavers and cleavers on flakes, and a structured core technology as early as 700 ka. The bifaces in the lower (older) level (n = 33 complete pieces, n = 835 total, see Fig 2 below) are produced mainly on thin millstone slabs (around 3–4 cm thick), and include both classic handaxes, bifacial tools and cleavers. They are diverse in shape and have different degrees of shaping intensity, with some tools showing a possible combination of the use of hard and soft hammer.

The bifacial tools are made by peripheral removals originating on the cutting edges. Sometimes the bifacial edges are opposed to a natural back resulting from the shape of the slab. Additional retouch on the edges is rare. The peripheral removals suggest that these tools were additions of independent edges on the blank. On the more intensively worked handaxes, three successive series of scars may be observed. The first is a series of deep and invasive removals using face-by-face or alternate shaping, with a second series of shorter and thinner removals managing the overall volume. Then final retouch is applied to parts of the sharp cutting edges and the tip. Edge angles vary between 50° and 80° for each category.

**Fig 3:**
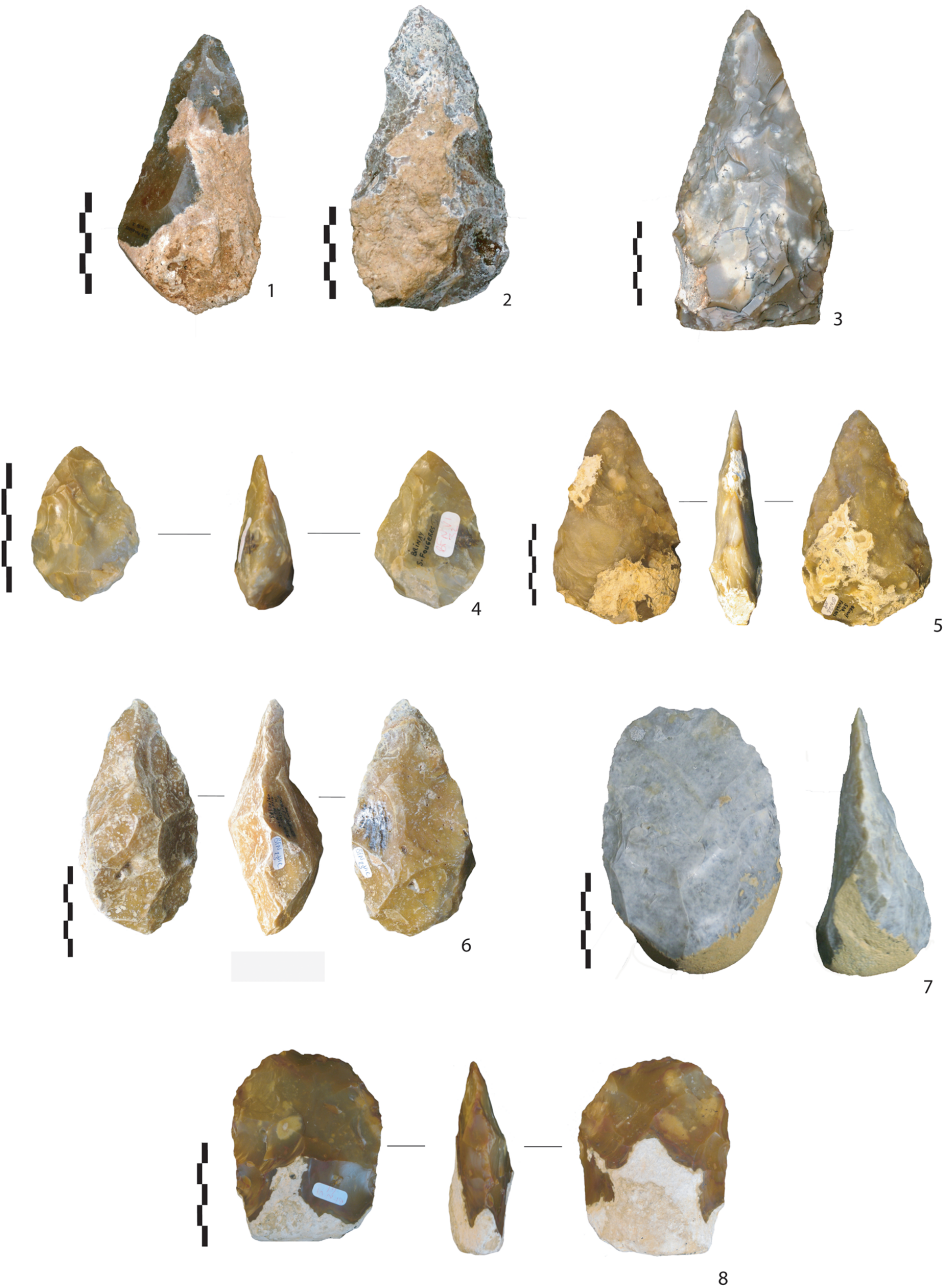
Handaxes from la Noira, upper level. Examples of the types of millstone and flint handaxes at the upper level at la Noira: bifaces with the bifacial upper part (1, 4), bifaces (2, 3, 7, 8) and bifacial cleavers (5, 6).

The upper level (n = 49 complete bifacial pieces, n = 645 total; see Fig 3 below) shows several important differences: first, both local millstone and secondary semi-local Cretaceous flint coming from a radius of 30 to 100 km to the southeast and west of the site) are employed as raw materials. Second, the bifaces are more intensively worked, and the use of soft hammer techniques is more common. Third, removals are much more numerous, covering the surfaces or the upper part of the biface (toward the tip). Sinuous and semi-sinuous edge profiles are virtually absent, and final retouches becoming more frequent, possibly indicating a better control of overall handaxe shape and a careful management of the bifacial volume and/or the tip.

#### Boxgrove

The site of Boxgrove is located in West Sussex, southern England, at the intersection of a chalk escarpment and a coastal plain adjacent to the English Channel. The coastal plain has been formed during the last 500 ka through marine erosion during interglacials coupled with tectonic uplift. This process has created a staircase sequence of wave-cut platforms. The Paleolithic site lies on the highest of these, ca. 40 m a.s.l. The Boxgrove paleolandscape is dated biostratigraphically to MIS 13 (ca. 480 ka) [43] and has been geologically mapped for over 26 km [44], making it one of the best-exposed Lower Paleolithic contexts in the world.

Abundant lithic and faunal materials has been recovered from over 90 locations on this landscape, at times showing remarkable temporal resolution, and largely free from post-depositional disturbances [45]. Most of the lithic material seems to originate in the production, maintenance, and retooling of handaxes [44] during butchery activities. Following extensive technological [46], refitting [47], and use-wear [48] studies, it has been suggested that bifaces were discarded at Boxgrove in a highly structured way [49], perhaps as hominins returned to the chalk escarpment which contained the fine-grained flint used as raw-material for the production of new handaxes [44]. This kind of discard pattern has been previously proposed for Olorgesailie members 1 and 7 in East Africa [50] and therefore represents a known behavior for the Acheulian.

The sample of complete (unbroken) handaxes in this article (n = 376) is an aggregate from all the excavations, and includes both published and unpublished material kept at Frank's House, British Museum, 56 Orsman Road, London N1 5QJ, United Kingdom of Great Britain and Northern Ireland (for inquiries to study the collection call +44 (0)20 7323 8100) (see also [51]). To the best of our knowledge, a complete description of the entire aggregate has not yet been published (permission to perform a morphometric study of all materials was given by Nick Ashton, Curator of Palaeolithic and Mesolithic collections, as well as by the excavator, Dr. Mark Roberts). Given the study design, which includes all the complete pieces, individual specimen numbers were not collected and cannot be rendered in full in this paper. Given its makeup, the Boxgrove aggregate represents an 'average' of handaxe maintenance and discard at the site which likely spans the entire period of occupation of the paleolandscape. For this reason, it can be used as a 'measuring stick' against which to evaluate the morphometric properties of the handaxes from la Noira.

### Symmetry quantification using continuous symmetry measure (CSM)

The continuous symmetry measure (CSM) method [52–54] was originally developed to study chemical phenomena related to molecular symmetry, such as chirality (asymmetry) and shape, and has been widely applied since then in chemistry [55–57] as well as other disciplines in natural science [58,59]. The first application of the CSM method in archaeology was the study of Saragusti et al. [14] in the context of handaxe symmetry. Machin et al. [22] have also used CSM on a set of experimental handaxes used to test the influence of symmetry on butchery effectiveness, although the actual computation method was not published alongside the data. In the following years, Saragusti et al. [60], as well as other researchers [30] applied the tangent curve method to study handaxe symmetry, obtaining results similar to those derived from the CSM method. It should be noted however that the tangent curve method was defined for the study of reflection symmetry and is missing the size normalization factor. In this respect, the CSM method is far more general.

The input for a CSM calculation are the actual coordinates *{****Q***_*k*_, *k = 1*,*2*,*…*,*N}* of a shape's vertices (here, created by the digitization of an outline) and the desired symmetry point group *G*. The algorithm generates a series of structures with the same number of vertices as the initial structure but with symmetry *G*. From this series, the selected final symmetric structure with coordinates *{****P***_*k*_, *k = 1*,*2*,*…*,*N}* is the configuration which minimizes the distance to the original structure as defined by Eq (1):

$$S\left(G\right)=100\times \frac{\sum_{k=1}^{N}{\left|Q_{k}-P_{k}\right|}^{2}}{\sum_{k=1}^{N}{\left|Q_{k}-Q_{0}\right|}^{2}}$$ (1)

**Fig 4:**
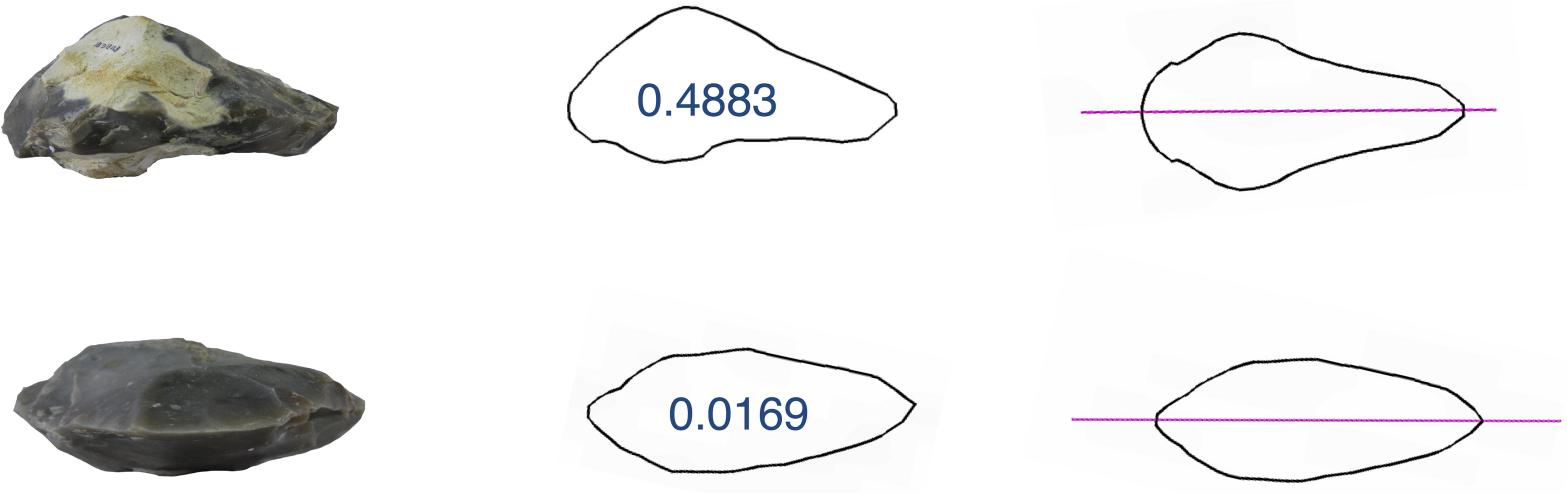
Examples of two handaxes (frontal views). Left: original photos, middle: extracted contours (numbers represent the S(Cs) values; right: reconstructed nearest symmetric shapes.

Here ***Q***_*0*_ is the center of mass of the original distorted structure. *S(G)* in Eq (1) is called the continuous symmetry measure. The denominator in Eq (1) is a mean square size normalization factor, which is introduced to avoid size effects. The CSM defined in Eq (1) is independent of the position, orientation, and size of the original structure. It is a global parameter, and therefore allows the comparison of various structures and various symmetries on the same scale. Eq (1) is a special distance function in that the target structure is unknown but is searched. This involves several minimizations, which are carried out both analytically and numerically over all possible permutations between the vertices of the original structure and vertices of its symmetric counterpart. The values of *S(G)* are between *0* (when the original structure is symmetric) and *100* when the nearest structure of *G* symmetry reduces to a point in space. All CSM calculations were performed with the CSM code of Avnir et al. [53,54]. For online calculations of CSM a website is freely available [61] at http://www.csm.huji.ac.il/new/ and http://telem.openu.ac.il/csm/ [62]. It is also possible to visualize the shapes in comparison to their nearest most symmetric version (see below Fig 4).

#### Permutation analysis

**Fig 5:**
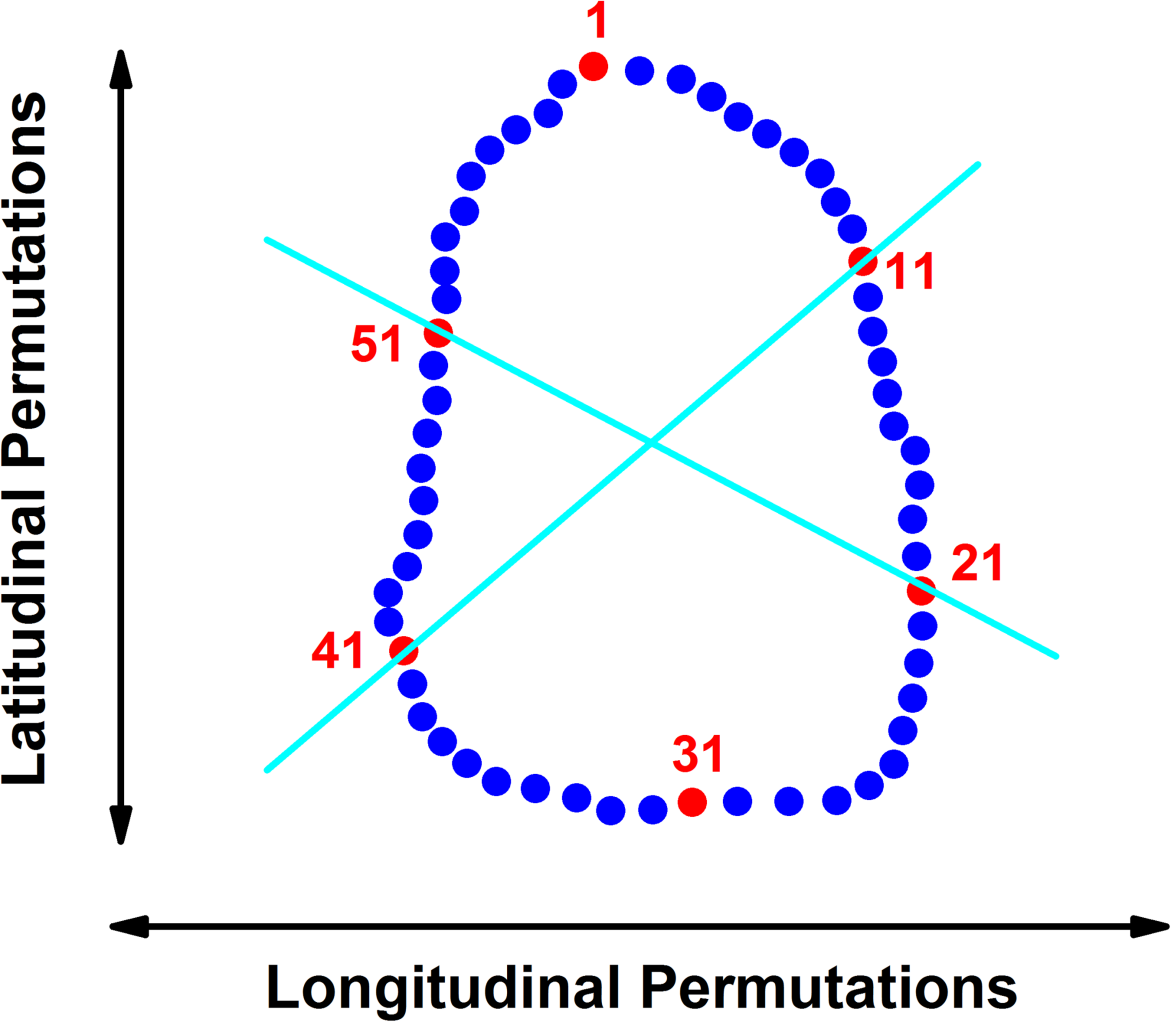
Latitudinal and longitudinal permutations of a contour. Illustration of a handaxe contour, showing how the latitudinal and longitudinal permutations are defined based on the orientation of the optimal symmetry line.

All analyses were performed using a 60-point digitized outline. The number of sampled points was pre-determined by the manual data collection of the Boxgrove handaxes for a different study [51]. For a closed shape of 60 vertices, there are 60 possible options to draw a symmetry line: 30 lines go through two opposing vertices (i.e., (1,31), (2,32),…(30,60)). The other 30 lines pass between two adjacent vertices and between two other opposing vertices on the other side (e.g., between (1,2) and (31,32), etc.). Each permutation was given a numerical index. Vertex 1 is defined in the sharpest edge of the handaxe. We mark permutations starting at vertex 11 through 20 as latitudinal permutations, with indices 1..20, and all other permutations starting at vertex 21 through vertex 40, as longitudinal permutations with indices 21..60. We note that permutations starting at vertex 41 through 50 are equivalent to the latitudinal permutations, while those starting at vertex 51 through 60 and 1 through 10 are equivalent to the longitudinal permutations. Fig 5 presents a handaxe with vertex labeling and the permutation definition.

### Shape description using Elliptical Fourier Analysis

Elliptical Fourier Analysis (EFA) is a method for parameterizing closed curves that is based upon Fourier series approximations of the x- and y-coordinates of outlines. The advantages of EFA over other Fourier methods include the lack of a need for equally spaced points, the fact that Fourier coefficients can be made independent of outline position, and the possibility of normalizing for size. EFA is used relatively often in paleontology and biology in general [63–69] including paleoanthropology [70–73], and has proven useful in quantifying curves lacking homologous landmarks. Fourier methods have been rarely used in stone tool analysis [51,60,74–80] but have seen a resurgence within a renewed interest in quantitative shape analysis of archaeological artifacts [19,21,79–92].

The data collection protocol employed here ([75,76] revised in [51]) consists of automatically extracting 2-dimensional coordinate data from digital photographs taken of each handaxe at a 90-degree angle, for three different views (named “Top” (also frequently known as “plan”), “Lateral” (“side”), and “Frontal” (“cross-section” or “transversal” or “tip-on”)). Raw coordinate data were then extracted from these photographs using scripts available at https://raduiovita.wordpress.com/software). Orienting handaxes in a consistent way is notoriously difficult [93], yet absolutely necessary for a quantitative study of shape. In this case, all artifacts were oriented with the tip to the right of the photograph, and the most convex side facing the camera. Further standardization (size and orientation) of the outlines was done by a full generalized Procrustes alignment using the “Momocs” package [94] for R [95]. The raw elliptical Fourier (EF) descriptors were passed on to a principal components analysis (PCA) for dimensional reduction.

### Quantifying the effect of reduction

The effect of reduction as a factor determining lithic shape variability has been the subject of major debates in the lithics literature (see [96,97]. for comprehensive reviews). While various geometric indices such as the GIUR [98,99] have largely solved the problem for flake tools, where blank shape and size is largely predetermined by platform attributes [100,101], the problem is more complicated in the case of shaped implements. Here, original, pre-resharpening blank shape and size are often unknown and very dependent on the type of production technique. For instance, Wilson and Andrefsky [102] noted that most of the mass lost in the total reduction of North American prehistoric bifaces was lost in production, not resharpening. For this reason, in the North American lithic literature, some tool reduction indices focus on edge maintenance [103]. Others, such as Johnson’s Thinning Index (JTI, which measures the ratio between plan view area and weight [104,105]) track reduction by looking at allometric relationships. Allometric effects are observed when a measure of overall size (such as weight) is compared with a particular linear measurement, such as length [51,75,106–108], or, indeed with a quadratic one, such as area (JTI). The fact that the targeted measurement changes non-proportionally with respect to size is used as both index of reduction and explanation of shape change [108–110]. In this paper, we rely on independent shape descriptors derived from elliptical Fourier Analysis and therefore, we chose to use Clarkson's [111] index of invasiveness for tracking reduction in the La Noira handaxes. This index, which ranges from 0 (unretouched) to 1 (completely covered by retouch scars) is highly correlated with (the square root of) mass lost due to reduction (be it by ‘thinning’ or resharpening, which are sometimes difficult to distinguish in Acheulian handaxe manufacture). Its applicability to both bifaces and unifacial tools, as well as its agnosticism toward reduction technique are among the reasons to choose it. Unfortunately, the Boxgrove data were collected much earlier for a different project and the index of invasiveness could not be calculated from photographs, because only one of the two faces was photographed per artifact. The La Noira data were also collected before the publication of a more recent index based on flake scar density [112], so its values were likewise not calculated. Size was measured as the centroid size of the respective view (top, lateral, or frontal, in mm), or when the whole artifact was taken into consideration, by the geometric mean of the three centroid areas. Because the density of flint is reasonably constant, metric-based measures of size can be used instead of mass.

## Results

### Symmetry is high and independent of technological factors

**Fig 6:**
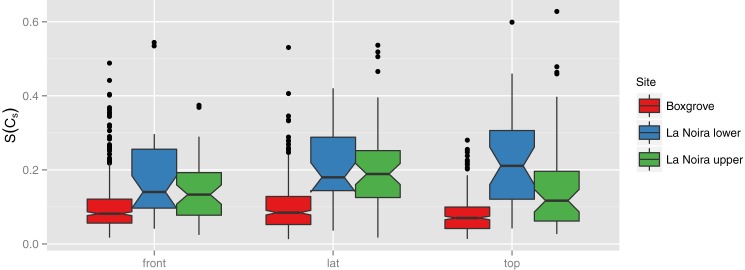
Summary of symmetry for each assemblage and view (cross-section). Note that Boxgrove handaxes are more symmetric in all views, although the difference is not large.

For each structure, S(C_s_) was calculated with respect to all possible permutations, and the permutation which gave the minimal S(C_s_) was chosen. The most important result is that S(C_s_) values are generally small regardless of the view (top, lateral, or frontal). This means that all handaxes have relatively high symmetry regardless of the site or specific permutations. However, the handaxes of la Noira are somewhat less symmetric than those from Boxgrove (see Fig 6 below). This is in particular true for the handaxes from the lower level at la Noira, which are the oldest and least symmetric of the three groups and include various sub-types of bifacial tools.

**Fig 7:**
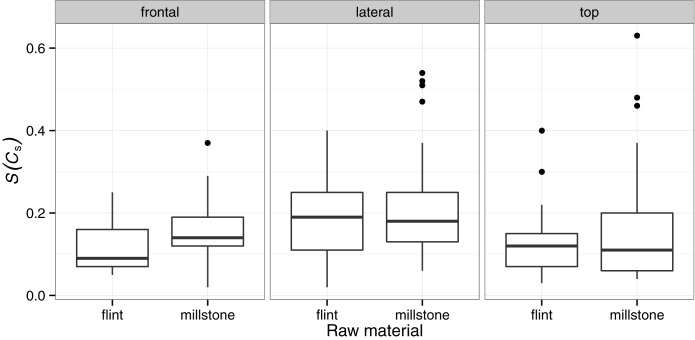
Variation in symmetry by raw material type.

Remarkably, symmetry is not affected by most technological factors. In the upper level at la Noira, we were able to compare the symmetry of the handaxes produced on millstone slabs with that of those worked in flint (see Fig 7 below). None of the three views showed statistically significant differences, indicating that hominins were able to work both materials with equal ease and applied the same technological and morphological features.

**Fig 8:**
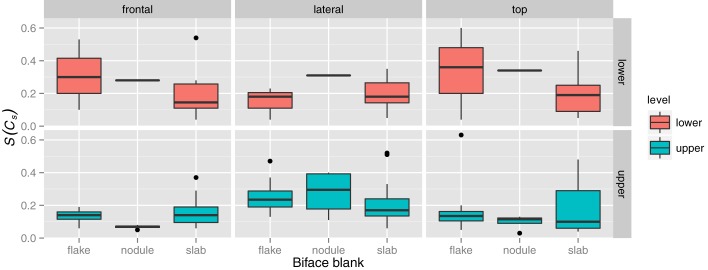
Variation in symmetry by biface blank type (i.e., the flake, nodule, or slab used to fashion the handaxe).

The type of blank used seems to also make little difference, although the few bifaces on large flakes, rather than those on slabs, are the most variable. This is possibly due to a limited shaping on the blank, but enough for managing the tool volume, including the two convergent cutting edges and the tip (Fig 8). Further, we do not detect any difference among handaxes with predominantly alternating, face-by-face, or bifacial reduction, indicating that form was maintained through a suite of different techniques. Unifacial reduction (face by face) did result in slightly more asymmetric frontal views (plano-convex tools) than those produced by bifacial retouch (alternate shaping) in the lower level at la Noira (t = 2.70, df = 25, p < 0.05). However, this was the only significant relationship between symmetry and shape detected, and it is expected, because unifacial reduction by definition affects one face more than the other.

Most surprisingly, S(C_s_) is not correlated with the degree of reduction as tracked by either size or Clarkson’s index of invasiveness [111]. This means that more heavily reduced handaxes are not more asymmetric than those at the beginning of their life-history (as tracked by these measures). Consequently, barring a non-random discard pattern, symmetry was either part of the design of the tools whatever their type, or a geometrically deterministic outcome of the reduction process (for example, a by-product of alternating the main flaked surface).

### The symmetry axis is 3-D longitudinal

**Fig 9:**
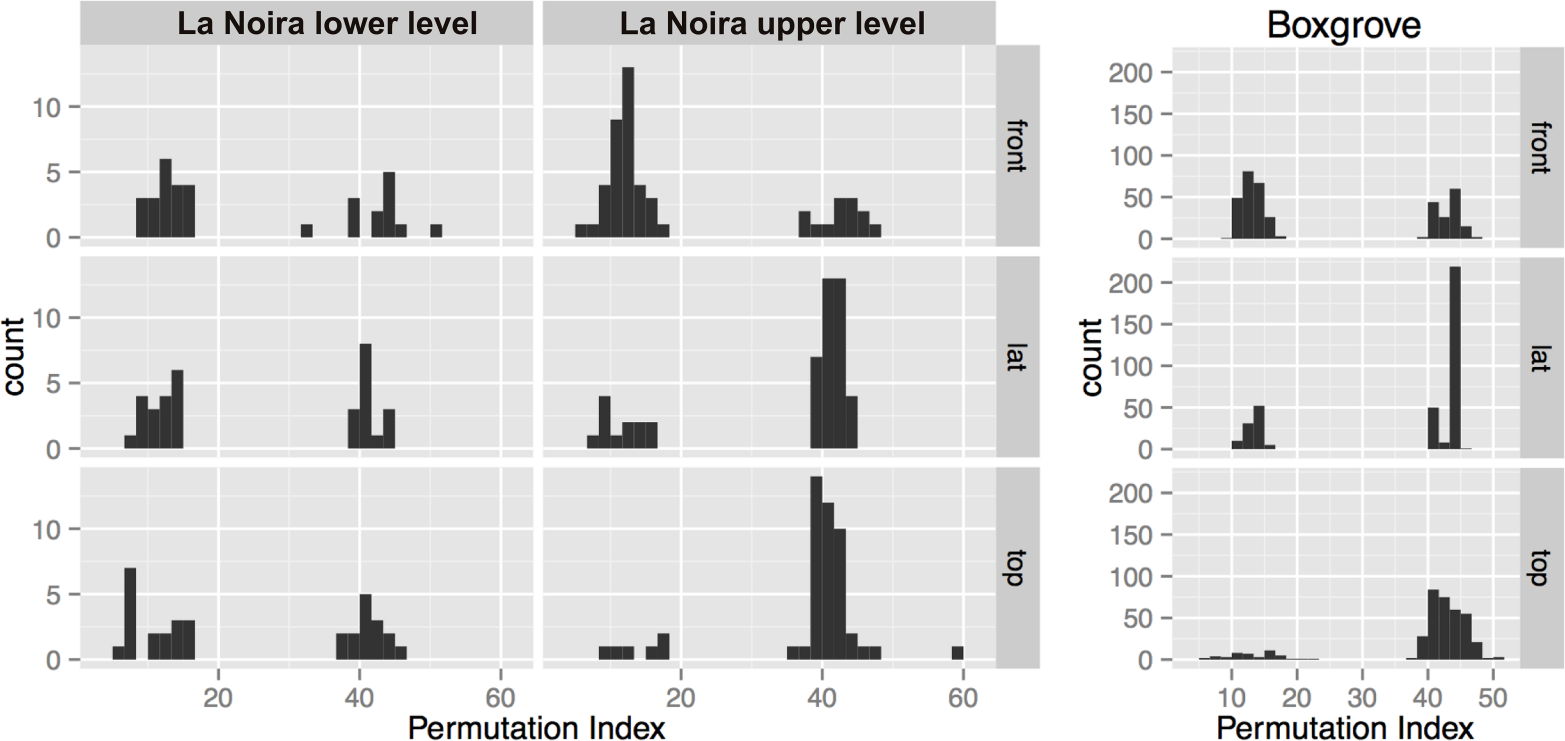
Permutation distribution for all assemblages and views. The graph shows a clear separation between latitudinal and longitudinal handaxes according to the placement of the axis of best symmetry.

As mentioned above, most handaxe contours have either a longitudinally or a latitudinally-placed symmetry line. In most studies to date, this is simply assumed to be the *intended* symmetry axis, but this must also be demonstrated. Fig 9 shows the distribution of permutations that give a minimum CSM value for the handaxes from both sites.

**Fig 10:**
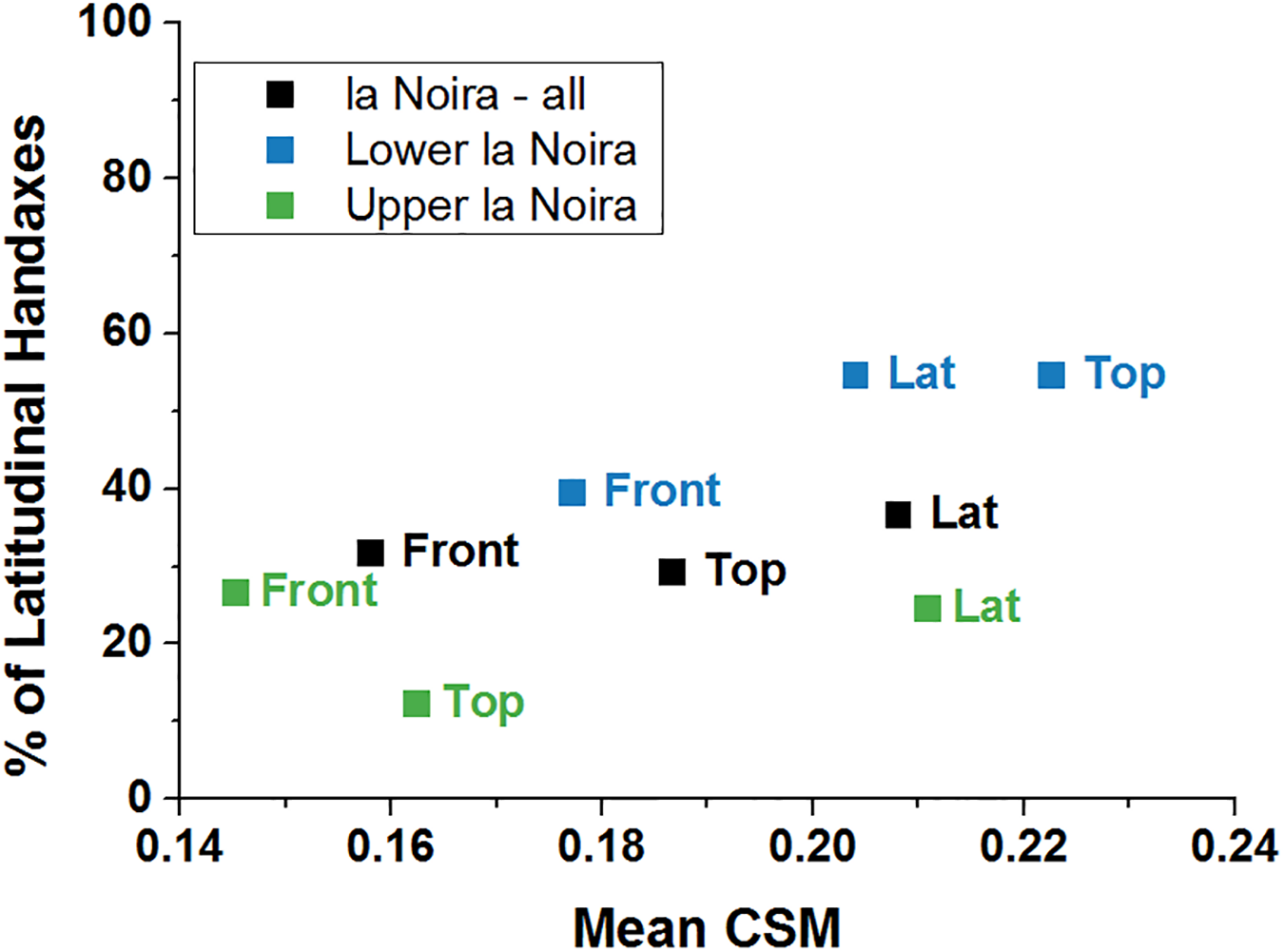
Percentage of latitudinal handaxes (where the line of best symmetry is perpendicular to the longest axis) at la Noira. Points represent averages per assemblage. Note the presence of many more latitudinal handaxes in the top view for La Noira lower level (almost 50%).

There is a clear separation between the two types of permutations, indicating that the main line of symmetry is either longitudinally or latitudinally oriented. In the top and lateral views, for both sites, the majority of handaxes have the oft-quoted and assumed longitudinal symmetry (along a line running from the 'tip' to the 'base', which are homologous in both the lateral and top views). However, looking at the frontal view, the number of handaxes with latitudinal symmetry increases for each assemblage (Fig 10). At la Noira upper level, this is quite extreme. However, due to the orientation of the frontal view at a 90° angle to both of the other views, a latitudinal symmetry here is equivalent with a longitudinal symmetry for the top and lateral view. We thus conclude that the orientation of the symmetry line is 3-D longitudinal for the majority of handaxes. Another interesting corollary of the prevalence of latitudinal symmetry in the frontal view is that symmetry in the top view takes precedence over face-to-face symmetry. In other words, whether or not by intention, the process that led to the production of symmetry in all of these handaxes focused not on maintaining what is generally called a 'volumetric' symmetry of the two faces, but rather a side-to-side, edge-focused one.

### Symmetry and shape as a function of discard and reduction

Because handaxe assemblages are time-averaged aggregates, it is important to regard each object as residing along a continuum of reduction. Therefore, no discussion of symmetry can be complete without a look at the well-known allometric changes that take place during handaxe reduction.

**Fig 11:**
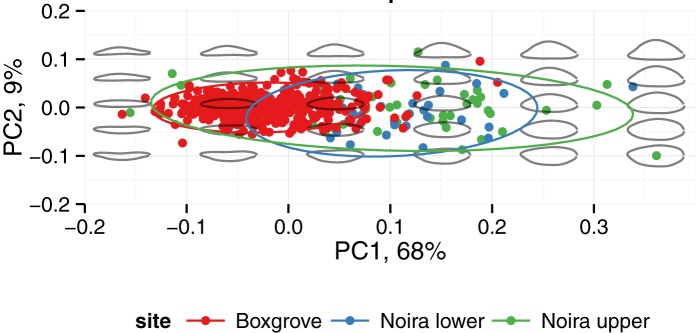
Visual representation of shape variation in the handaxes from la Noira and Boxgrove (frontal view).

**Fig 12:**
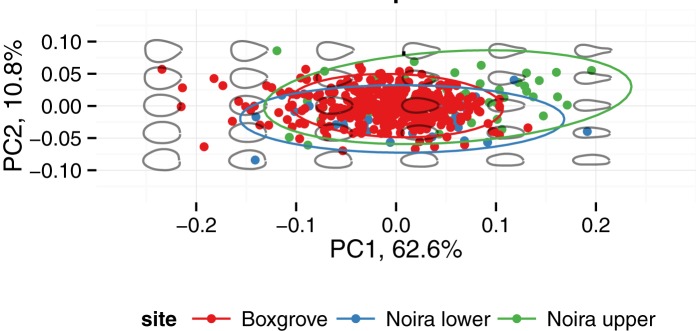
Visual representation of shape variation in the handaxes from la Noira and Boxgrove (top view).

**Fig 13:**
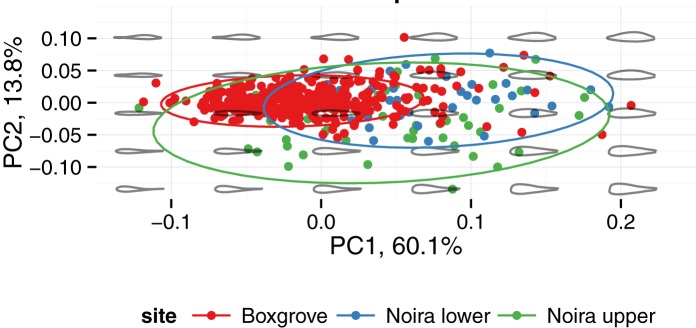
Visual representation of shape variation in the handaxes from la Noira and Boxgrove (lateral view).

Handaxe shape is well characterized by the first three principal components of the 8 harmonics of Elliptical Fourier coefficients. Here we lumped the assemblages from the lower (n = 33) and upper (n = 49) levels into one (n = 82), because the sample sizes would otherwise be too small for PCA. As reported elsewhere for handaxes [51], PC 1 (63% of the variance for the top view; 60% and 68% for the lateral and respectively, frontal views) describes the degree of elongation of the piece. Because of the different 3-D orientation, in the *frontal view* (see Fig 11 below), this same PC 1 corresponds to the flatness (or refinement, as it is often called in handaxe shape studies) of the artifact. In the *top view* (see Fig 12), PC 2 (13% of variance) describes the left-right orientation of the longest edge and PC 3 (10% of the variance) describes the location of the point of maximum width (in terms of the distance from the tip). In the *lateral view* (see Fig 13 below), PC 2 (11% of variance) describes the location of the maximum thickness (in terms of the distance from the tip). Finally, in the *frontal view* (Fig 11), PC 1 (68%) describes the refinement (relative thickness, see above), whereas PC 2 roughly describes the plano-convexity of the piece.

For la Noira, PC 1 (elongation) in the top view is weakly correlated with PC 1 in the lateral view (lower level: *r* = 0.51, *t* = -3.3, df = 31, *p* = 0.003; upper level: *r* = 0.34, *t* = -2.5, df = 47, *p* = 0.016), and also with refinement (lower: *r* = 0.48, *t* = 3.0, df = 31, *p* = 0.005; upper: *r* = 0.35, *t* = 2.6, df = 47, *p* = 0.014). This suggests that, overall, more elongated specimens were also comparatively flatter/more refined, i.e., that volumes were generally managed whole.

One of the surprising trends present in the handaxes from both levels at la Noira is that elongation, as represented by PC 1 of the top and lateral views, is *not correlated with reduction*. This is in contrast to the well-known trend linking ovate and pointed handaxes through tip reduction [109], which is weak, but statistically significant at Boxgrove [51] and better demonstrated in other Acheulian handaxe data published before [108–110,113]. Shipton and Clarkson [112] were not able to replicate it for Boxgrove in their geometric morphometric study, but their sample size was less than 10% of that used by Iovita and McPherron [51]. This allometric pattern[106,107] can be described visually as the progressive rounding of the entire handaxe contour as size decreases through the progressive removal of the sharp tip and is one of the strongest results in handaxe shape studies overall, despite differences in its interpretation [106,107]. Only the upper level at la Noira shows a weak correlation between PC 1 of the front view (which tracks refinement) and size (*r* = 0.33, *t* = 2.4, df = 47, *p* = 0.02), i.e., the smaller handaxes are also relatively flatter in the transversal (frontal) cross-section, which may be a reflection of a combination of using millstone slabs or large flakes as blanks. The millstone handaxes are somewhat more refined than the flint ones, but the difference is not significant. This could, however, be due to sample size.

There could be several reasons for the differences between shape changes at la Noira and the documented Acheulian pattern of tip reduction. Two of the most obvious are the different discard patterns and the relatively small sizes of the la Noira samples. One of the assumptions (simplifications) typically made when analyzing handaxe shape is that discard is random with respect to reduction stage. Because of the sample size of the aggregate (n = 376) and the large area from which it originates, this assumption is probably safe with Boxgrove. However, in the case of la Noira, it appears to be violated, with the upper level having a large number of handaxes of various sizes that have the maximum index of invasiveness. The majority of these handaxes are made in flint (the Clarkson Index of invasiveness for flint handaxes is significantly higher than for those made in millstone, Wilcoxon *W* = 358.5, *p* < 0.05). These flint handaxes were probably more intensely worked because the material was likely brought from more distant outcrops, while millstone bifaces could be easily discarded in exchange for fresh preforms. The regression of geometric size on the index of invasiveness is still significant for both levels of la Noira, but the coefficients of determination are low (*R*^2^ = 0.18, *p* < 0.05 for the lower level and *R*^2^ = 0.15, *p* < 0.01 for the upper level). Therefore, using size to track reduction is marginally legitimate, although having an independent measure such as the index of invasiveness provides more nuance.

## Discussion and conclusions

The main result of our analyses is that handaxes from the lower layers at la Noira, which are at least 700 ka old, already exhibit a high degree of longitudinal symmetry that is largely invariant of a variety of factors usually thought to affect the ability of prehistoric hominins to work stone, such as raw material quality and reduction technique, as well as reduction stage. Although on average, the handaxes from the upper level (ca. 500ka old) are somewhat more symmetric than the ones from the lower level, the difference is very small, and is also dependent on the stage of reduction and the raw material. In practice, this means that different assemblages may exhibit different average symmetry values depending upon the discard distribution, which is important to know when considering the evolution of either cognitive capacity or manual dexterity. It is likely that the shorter duration of occupation at Boxgrove and its different function played a role in the symmetry signal, the expectation being that Boxgrove, one of the best-known ‘slices of time’ in the Paleolithic, would display more finely finished handaxes. If La Noira was indeed a ‘quarry’, this would further bias the symmetry signal by taking away some of the ‘finished’ handaxes for tasks to be performed out in the landscape. This ‘unfair’ comparison yielding relatively small differences between the two sites reinforces how symmetric the La Noira specimens actually are. In terms of average values, both levels at la Noira are less symmetric than the Boxgrove assemblage, but again, these differences are very small, and several of the la Noira lower level handaxes can be found within the quartile with the lowest *S(C*_s_*)* values. Therefore, we can conclude that, by 700ka, Acheulian toolmakers already possessed considerable abilities to shape stone into symmetric shapes, whatever the causes of this symmetry may have been.

One of the major questions arising from this is why handaxes are symmetric at all. In other words, if maintaining symmetry is technically challenging, why did hominins invest into this property? This question is not trivial: while cognition and manual dexterity represent limiting factors to the production of symmetric objects, their function (or, rather purpose) determines the requirements as well as the parameters which are left free to vary. In an attempt to answer this question, and passing over some non-utilitarian explanations for stone handaxes [2,114,115], (for a recent review of bone handaxes, see [116]) which have the double disadvantage of being untestable and, in some cases misguided in their assumptions (for counter-arguments, see [81,117,118]), we are left with direct forensic evidence of the past use of handaxes. The few use-wear and studies of Acheulian handaxes [48,119–122] have revealed surprisingly little precise information on their uses beyond 'butchering activities'. Woodworking is seen as the main activity by comparatively fewer authors [123,124]. However, it is difficult to generalize about handaxe use to the extent that would be necessary in order to complement the plethora of morphometric and technological studies on handaxe shape. Several recent experimental studies have approached the question of symmetry from a functional efficiency point of view [22,125], shedding some light on why symmetry *might* be important for efficient butchery, although without stating that it is a necessary property of this type of implement. However, without supporting data on how archaeological handaxes were *actually used*, much of this discussion remains speculative.

The other main question raised by the results presented here is if there is a trend toward increasing symmetry and when that trend might have begun. Unfortunately, a statistically valid comparison between the values presented here and those published by Saragusti et al [14] whose sample stretched back further in time is not directly possible. Although Saragusti et al. also used the CSM method (with a slight numerical difference in the normalization constant), their sample sizes are too small to perform any statistical tests. However, it is clear that some of their CSM values > 1 displayed by the assemblages from Ubeidiya and Gesher Benot Ya'aqov show considerable more asymmetry than either Boxgrove or la Noira, which are more similar to the values reported from Maayan Baruch. Taken together, the ages of all the sites studied using CSM (Levant and Europe), it does appear that there is indeed a trend toward increasing average symmetry through time. However, the starting point for the transition appears to be at the beginning of the Acheulian outside of Africa rather than at the arrival in Europe. Since contradicting evidence to a trend toward standardization and symmetry (using more subjective criteria) is offered by McNabb and Cole [18], who point out that some of the later handaxes are less symmetric than the early examples, this matter needs to be settled in a subsequent study that includes more assemblages. Either way, evidence of a lesser degree of symmetry in later handaxes does not invalidate results that show a high degree of symmetry being imposed on specimens from the early part of the Acheulian, as we have done here. Therefore, we can estimate the minimum date for the documented appearance of an ability to control form–independently of its possible motives.

The two questions reviewed briefly above are connected. One of the difficulties of comparing across such great chronological expanses and using only three data points is that, without knowing the exact function of individual objects submitted to morphometric analyses, it is possible to compare apples with oranges. Even on the level of very coarse distinctions, the extent to which bifaces were used primarily as edge-tools versus cores should play a major role in how symmetric they were, regardless of raw material or knapping ability. A functional difference obscured by similar shape has been previously shown both qualitatively through a number of techno-functional studies [126,127], as well as quantitatively through morphometrics [51]. That is, we may be lumping very different objects under the term “biface” or “handaxe”, and this could result in inappropriate conclusions about motor and cognitive abilities. If the earliest bifaces in the African Acheulian developed from core-tools, it makes sense that symmetry in these tools would be reduced in comparison with fully worked, retouched tools from the later Acheulian. For instance, Beyene et al. [13] noted that after 1.0 Ma, the bifaces at Konso typically exhibit more flake scars, and this is correlated with an increase in standardization (although 'standardization' was not objectively quantified). This effect may be amplified by the fracture properties and grain size of the raw materials. Our data suggest that raw material quality does not significantly influence the ability of Acheulian hominins to shape very symmetric handaxes. It is the availability rather than the ease of shaping which may determine on average how symmetric handaxes are when they enter the archaeological record. Nevertheless, both millstone and the flints available in most of the European Acheulian sites are demonstrably different in their flaking properties from the volcanic or quartzitic rocks that dominate the Early Stone Age record in Africa. To the best of our knowledge, a comparative study that takes this factor into account has not yet been undertaken.

In conclusion, the morphometric analysis performed at la Noira, compared to Boxgrove, is supported by the morpho-technological analysis of the handaxes [29]. At the 700 ka old lower level from la Noira, handaxes, accompanied by other bifacial tools and cleavers, are already fully developed and display a high degree of symmetry, even when compared with the short-occupation accumulation from the Boxgrove paleolandscape. At 500 ka, in the upper level of la Noira, symmetry is slightly more developed and the raw material gathering perimeter increases in size, although the differences are relatively small. Our study also has implications for dating the onset of bifacial technologies in Europe. Because of a lack of clear evidence of transitional assemblages leading up to bifacial technology in Europe, most current hypotheses propose the introduction of an a full-blown bifacial technology which had developed elsewhere [27–29]. Discoveries made over the past decade confirm that the onset of bifacial technology in Western Europe occurred much earlier than previously thought, namely between 1 Ma and 500 ka. During this period, sites with bifacial technology are still rare, their number increasing considerably only after 500 ka and the MIS 12 glaciation, both in the North and the South of Europe. So, the appearance of handaxes in Europe must have taken place in parallel to the existence of “classical” Acheulian facies in East Africa from 1 Ma to 500 ka such as at Olorgesailie, Garba XII, Isenya or Isimila [128–132]

Therefore, many questions regarding the origin and significance of such assemblages remain unresolved. Several different scenarios appear possible:

1. these early bifacial industries may be of local origin in some areas;
2. core-and-flake technology may have persisted, with some changes, after the arrival of bifacial technology but would have incorporated technological innovations due to contact with new hominin groups or ideas [133]; or
3. the diversity of traditions may be due to the successive arrivals, slow or rapid (with extinction or not), of new technological traditions and know-how coming from Asia, the Levant and/or East Africa where similar assemblages existed.

In the last case, bifacial technology may have no connection to the local substratum. Our results on handaxe symmetry would lend support to this third hypothesis. The chronological gap observed in Western Europe between 700 ka old assemblages and the more commonly seen 500 ka old assemblages (e.g., Boxgrove, la Noira upper level) might reflect a real depopulation event, with the occupation from the upper level at La Noira linked with the later arrival of new groups with a better control of bifacial technology after 500 ka. However, at this moment, the data are too sparse to test such hypotheses rigorously. Future work integrating more lithic series of various ages from sites such as La Boella, Arago, Notachirico, Carriere Carpentier, Maids Cross Hill, High Lodge, Warren Hill, Swanscombe, and Cagny La Garenne, and using the same methods would greatly enhance our ability to ask the bigger questions about settlement, cultural exchanges, and technological transfer in the Acheulian.
